# Differential effectiveness of COVID‐19 health behaviors: The role of mental health conditions in mask‐wearing, social distancing, and hygiene practice

**DOI:** 10.1002/hcs2.60

**Published:** 2023-07-16

**Authors:** Yusen Zhai, Xue Du

**Affiliations:** ^1^ Department of Human Studies The University of Alabama at Birmingham Birmingham Alabama USA; ^2^ Heersink School of Medicine The University of Alabama at Birmingham Birmingham Alabama USA

**Keywords:** effectiveness, health behaviors, COVID‐19 infection, mental disorders

## Abstract

**Background:**

Mental health conditions are known to increase susceptibility to infectious diseases, including coronavirus disease 2019 (COVID‐19). Health behaviors play a crucial role in mitigating this susceptibility. We aim to examine the differential effectiveness of COVID‐19 preventive health behaviors among individuals, considering the presence or absence of specific mental health disorders.

**Methods:**

Multivariable logistic regression with interaction terms was performed to examine whether associations between adherence to health behaviors and COVID‐19 infection were conditional on depression, anxiety, or eating disorders in a national sample of adults (*N* = 61,891) from 140 US universities, 2020–2021.

**Results:**

Adjusting for age, race/ethnicity, and gender/sex, the effectiveness of mask‐wearing was significant and comparable among individuals with and without depression, anxiety, or eating disorders. Social distancing provided significantly less protection among individuals with depression, anxiety, or eating disorders. Hygiene practice provided significantly less protection among individuals with anxiety.

**Conclusion:**

Mask‐wearing is robustly effective in the prevention of COVID‐19 among individuals. However, social distancing and hygiene practice provide less significant protection among individuals with certain mental health conditions, suggesting the importance of prioritizing these individuals for additional preventive measures (e.g., vaccines targeting variants) and mitigation strategies (e.g., financial assistance, targeted mental health care, health education).

**Abbreviations**
*CI*
: confidence interval
COVID‐19: coronavirus disease 2019
HIV: human immunodeficiency virus
OR: odds ratio
SARS‐CoV‐2: severe acute respiratory syndrome coronavirus 2

## 1 INTRODUCTION

People with certain mental disorders are at greater risk for the coronavirus disease 2019 (COVID‐19) infection and related morbidity and mortality [1]. Historically, people with mental health conditions are more vulnerable to infectious diseases, such as human immunodeficiency virus (HIV) and seasonal influenza, enduring a more severe infection and clinical outcomes [2]. Before the COVID‐19 outbreak, over half of the US population experienced at least one diagnosable mental disorder at some stage during their life, such as depression or anxiety [3]. Given the increased vulnerability to COVID‐19 infection among people with mental health issues, more attention should be paid to this population to promote their health and longevity [1]. In addition to vaccination, COVID‐19 preventive health behaviors have played a crucial role in the prevention of COVID‐19 transmission in the general public [4], but the real‐world effectiveness of these health behaviors may have been different among individuals with mental disorders compared to those without. This study aimed to compare the real‐world effectiveness of health behaviors (i.e., mask‐wearing, social distancing, hygiene practice) adopted by individuals with and without specific mental disorders.

COVID‐19 preventive health behaviors remain essential to protecting people from COVID‐19 infection, given the ongoing concerns over vaccine breakthrough infections [5]. Mask‐wearing, social distancing, and hygiene practice can help manage SARS‐CoV‐2 transmission in the general population [4]. Meanwhile, individuals with mental health conditions are more likely to struggle with socioeconomic risk factors (e.g., poverty, overcrowded housing, lower health literacy [1]), which may put strain on the protective effects of health behaviors adopted by these individuals to reduce their risk of COVID‐19 infection. Thus, it is crucial to compare the effectiveness of COVID‐19 preventive health behaviors when adopted by different individuals to inform clinical practices and public health policies. So far, little is known about the difference in the effectiveness of these health behaviors adopted by individuals with and without specific mental disorders in real‐world settings. In this present study, we hypothesized that associations between adherence to health behaviors and COVID‐19 infection would be conditional on depression, anxiety, or eating disorders.

## 2 METHODS

### 2.1 Participants

This multicampus study was approved by the Institutional Review Board of the University of Alabama at Birmingham. Data were from Healthy Minds Network which surveyed a random sample of adults from 140 US universities from September 2020 to May 2021. The response rate was 14% in fall 2020 and 15% in winter/spring 2021, which is typical to a large‐scale online survey method [6]. Sample weights were calculated and used to adjust nonresponse based on institutional data, including sex, race/ethnicity, academic level, and grade point average. Weights are greater for participants from underrepresented groups to ensure that estimates properly represent the whole population at each institution.

### 2.2 Measures and covariates

COVID‐19 infection status was the binary outcome variable. Key independent variables were mental health conditions (i.e., clinically significant depression, anxiety, eating disorders) and adherence to health behaviors (i.e., mask‐wearing, social distancing, hygiene practice). Aligned with existing research methods [7], the variable representing adherence to mask‐wearing (“How often do you wear a facemask in public when it is required?”) was a continuous variable ranging from 1, *Never*, to 5, *All the time*. The variables representing adherence to social distancing (“How closely have you been following recommendations for social/physical distancing [keeping a six‐foot distance between yourself and others in public, avoiding gatherings of 10 or more people, and avoiding non‐essential trips outside your home]?”) and adherence to hygiene practice (“How closely have you been following recommendations for hygiene practices [frequent hand washing; avoiding touching your eyes, nose, and mouth; and disinfecting surfaces]?”) were continuous variables ranging from 1, *Not at all*, to 4, *Very closely*. Based on previous research [8], covariates included age, race/ethnicity, and gender/sex.

### 2.3 Data analysis

We performed logistic regression with interaction terms to compute adjusted odds ratios (aORs) and 95% confidence intervals (*CI*s) to examine whether associations between adherence to health behaviors and COVID‐19 infection were conditional on mental disorders. In stratified logistic regression models by mental disorders (clinically significant depression, anxiety, or eating disorders) and adherence to health behaviors (mask‐wearing, social distancing, or hygiene practice), the variables that represented mental disorders were binary variables, with “1” indicating a specific mental diagnosis.

As an abbreviated illustration of the modeling approach using a logistic regression framework including the variables previously described, the logistic model with interaction can be written as follows:

(1) $$\mathrm{logit}\;Y_{i}=\beta_{0}+\beta_{1}\;\mathrm{mental}\;{\mathrm{disorder}}_{i}+\beta_{2}\;\mathrm{preventive}\;{\mathrm{measure}}_{i}+\beta_{3}\;\mathrm{mental}\;{\mathrm{disorder}}_{i}\times \mathrm{preventive}\;{\mathrm{measure}}_{i}+\beta_{4}\;{\mathrm{covariates}}_{i}+\epsilon_{i}.$$

In this equation, *i* indexes participants. *Y*_*i*_ refers to the study outcome of interest (i.e., COVID‐19 infection status). *Covariates* indicate the demographic characteristics of participants. *β*s are the estimated effect of one unit change in corresponding variables on the log odds of *Y*. The coefficient (i.e., *β*_3_) of the interaction term, as the primary variable of interest, is the estimator of the effectiveness of adherence to one preventive measure on the log odds of *Y* moderated by one mental disorder. A significant interaction term indicates that the association between COVID‐19 infection and adherence to a preventive measure is conditional on a mental disorder.

In each model, when the interaction term was significant, the coefficient (i.e., *β*_2_) was the estimated effect of a one‐unit increase in adherence to a specific preventive measure on the log odds of acquiring COVID‐19 infection among participants *without* a specific mental disorder. To obtain estimated effects among participants *with* mental disorders, we again performed logistic regression with reverse‐coded binary variables representing mental disorders, with “0” indicating a specific mental diagnosis. At this time, the coefficient (i.e., *β*_2_) was the estimated effect of a one‐unit increase in adherence to a specific preventive measure on the log odds of acquiring COVID‐19 infection among participants *with* this specific mental disorder. A two‐sided *p* < 0.05 was considered statistically significant. Statistical analysis was conducted with SPSS version 26.

## 3 RESULTS

A total of 61,891 adults participated (Table 1). Results (Table 2) showed that a one‐unit increase in adherence to mask‐wearing, social distancing, or hygiene practice was significantly associated with lower odds of COVID‐19 infection in *both* individuals with and without clinically significant depression, anxiety, or eating disorders. The association between adherence to mask‐wearing and COVID‐19 infection was not conditional on clinically significant depression, anxiety, or eating disorders.

**Table 1:** Demographic characteristics of the sample, 2020–2021, United States (*N* = 61,891).

Characteristic	Participants, no. (%)^a^
Race/ethnicity	
American Indian/Alaskan Native	987 (2.0)
Asian	6744 (8.6)
Black/African American	6465 (12.2)
Latinx	4289 (8.3)
Native Hawaiian/Pacific Islander	193 (0.3)
Middle Eastern/Arab	782 (1.0)
White	41,637 (66.3)
Additional race	675 (1.3)
Sex	
Female	44,664 (60.4)
Male	17,151 (39.5)
Intersex	18 (0.03)
Gender	
Woman	42,703 (57.7)
Man	16,737 (38.5)
Transgender man	224 (0.4)
Transgender woman	110 (0.2)
Queer	516 (0.8)
Additional gender	1353 (2.0)
History of COVID‐19 infection	16,178 (27.3)
Clinically significant depression	13,260 (21.2)
Clinically significant anxiety	10,339 (16.4)
Eating disorders	7676 (11.7)

Abbreviation: COVID‐19, coronavirus disease 2019.

^a^ Sample sizes are unweighted, and percentages are weighted to be representative of the population at each institution.

**Table 2:** Multivariable logistic regression models showing associations of COVID‐19 Infection with mental disorders and COVID‐19 health behaviors.^a^

Variable	COVID‐19 infection	
	aOR (95% *CI*)^b^	*p* Value
*Model 1*		
Without clinically significant depression		
Mask‐wearing	**0.76 (0.74–0.79)**	<0.001
Social distancing	**0.69 (0.67–0.71)**	<0.001
Hygiene practices	**0.83 (0.81–0.86)**	<0.001
With clinically significant depression		
Mask‐wearing	**0.72 (0.68–0.77)**	<0.001
Social distancing	**0.74 (0.71–0.78)**	<0.001
Hygiene practices	**0.87 (0.83–0.92)**	<0.001
Interaction (clinically significant depression × preventive measure)		
Mask‐wearing	0.95 (0.88**–**1.02)	0.13
Social distancing	**1.07 (1.01–1.13)**	0.01
Hygiene practices	1.05 (0.99–1.12)	0.13
*Model 2*		
Without clinically significant anxiety		
Mask‐wearing	**0.76 (0.73–0.78)**	<0.001
Social distancing	**0.69 (0.67–0.71)**	<0.001
Hygiene practices	**0.82 (0.79–0.84)**	<0.001
With clinically significant anxiety		
Mask‐wearing	**0.75 (0.70–0.80)**	<0.001
Social distancing	**0.78 (0.74–0.82)**	<0.001
Hygiene practices	**0.89 (0.83–0.95)**	0.001
Interaction (clinically significant anxiety × preventive measure)		
Mask‐wearing	0.99 (0.92**–**1.07)	0.80
Social distancing	**1.13 (1.07–1.20)**	<0.001
Hygiene practices	**1.09 (1.01–1.17)**	0.02
*Model 3*		
Without eating disorder		
Mask‐wearing	**0.75 (0.73–0.77)**	<0.001
Social distancing	**0.70 (0.68–0.72)**	<0.001
Hygiene practices	**0.83 (0.81–0.86)**	<0.001
With eating disorder		
Mask‐wearing	**0.80 (0.73–0.87)**	<0.001
Social distancing	**0.81 (0.76–0.86)**	<0.001
Hygiene practices	**0.88 (0.82–0.95)**	0.001
Interaction (eating disorder × preventive measure)		
Mask‐wearing	1.06 (0.97**–**1.16)	0.20
Social distancing	**1.16 (1.09–1.24)**	<0.001
Hygiene practices	1.06 (0.97**–**1.14)	0.18

*Note*: Bold font indicates statistical significance.

Abbreviations: aOR, adjusted odds ratio; *CI*, confidence interval; COVID‐19, coronavirus disease 2019; GPA, grade point average.

^a^ Sample weights were used to adjust nonresponse based on institutional data on sex, race/ethnicity, academic level, and GPA.

^b^ Adjusted for age, race/ethnicity, and gender/sex.

However, associations between adherence to social distancing and COVID‐19 infection were conditional on clinically significant depression, anxiety, and eating disorders respectively. A one‐unit increase in adherence to social distancing was significantly associated with only 26%, 22%, and 19% lower odds of acquiring COVID‐19 in individuals with clinically significant depression, anxiety, or eating disorders, respectively, but 31%, 31%, and 30% lower odds of acquiring COVID‐19 in individuals without that mental health condition.

The association between adherence to hygiene practice and COVID‐19 infection was only conditional on clinically significant anxiety. A one‐unit increase in adherence to hygiene practice was significantly associated with only 11% lower odds of acquiring COVID‐19 in individuals with clinically significant anxiety, but 18% lower odds of acquiring COVID‐19 in individuals without that mental health condition.

## 4 DISCUSSION

Results revealed that the real‐world effectiveness of mask‐wearing was significant and comparable among individuals with and without depression, anxiety, or eating disorders. The findings suggest that mask‐wearing is an effective and robust preventive measure regardless of one's certain mental health conditions, reinforcing the importance of mask‐wearing to reduce the risk of COVID‐19 infection across individuals [7].

Notably, the results showed that although adherence to social distancing was significantly associated with lower odds of COVID‐19 infection across individuals, social distancing provided significantly less protection against COVID‐19 among individuals with depression, anxiety, or eating disorders. It is possible that socioeconomic risk factors (e.g., low socioeconomic status, overcrowded housing/communities [1]) might have refrained some people with certain mental disorders from avoiding crowded areas, though they strove for social distancing. Additionally, the results revealed that hygiene practice was significantly associated with lower odds of COVID‐19 infection across individuals; however, hygiene practice provided significantly less protection among those with anxiety. A possible explanation is that although people with anxiety attempted to adhere to hygiene practice, they might have more unconscious face‐touching than those without anxiety, which had increased their risk of COVID‐19 infection [9]. The findings underscore the importance of prioritizing individuals with mental health issues for additional preventive measures (e.g., vaccines targeting variants) and mitigation strategies (e.g., financial assistance, targeted mental health care, health education) to attenuate their risk of infection during any resurgence in the transmission of COVID‐19 [1].

This study has limitations. First, the retrospective, self‐reported data might introduce the possibility of recall bias; however, previous research shows that it is very unlikely to invalidate the importance of the findings [7, 10]. Second, this study is observational; thus, unobserved confounding factors might bias results. Third, the results might not be generalized beyond this current population. The response rate (14% in fall 2020 and 15% in winter/spring 2021) of the survey might introduce nonresponse bias, although sample weights were used to adjust nonresponse. Despite these limitations, this current study provides empirical evidence of the differences in real‐world effectiveness of COVID‐19 health behaviors adopted by individuals with and without specific mental disorders through a large national sample.

## AUTHOR CONTRIBUTIONS

**Yusen Zhai**: Conceptualization (lead); formal analysis (lead); investigation (lead); methodology (lead); project administration (lead); software (lead); validation (lead); writing—original draft (lead); writing—review and editing (lead). **Xue Du**: Investigation (supporting); project administration (supporting); validation (supporting); writing—review and editing (supporting).

## CONFLICT OF INTEREST STATEMENT

The authors declare no conflict of interest.

## ETHICS STATEMENT

Authors assert that all procedures contributing to this work comply with the ethical standards of the relevant national and institutional committees on human experimentation and with the Helsinki Declaration of 1975, as revised in 2008. All procedures involving human participants were approved by the institutional review board (IRB‐300008474) of the University of Alabama at Birmingham.

## INFORMED CONSENT

Written informed consent was obtained from all participants. Survey data are deidentified to preserve participants' anonymity.

## Data Availability

The data that support the findings of this study are openly available upon request in The Health Minds Network at https://healthymindsnetwork.org.
